# Complete Genome Sequence of *Microbacterium* sp. Strain 10M-3C3, Isolated from an Extremely Phosphorus-Poor Lake

**DOI:** 10.1128/MRA.01649-18

**Published:** 2019-01-24

**Authors:** Priscilla Hempel, Jessica L. Keffer, Olga Shevchenko, Cynthia Henny, Shawn W. Polson, Julia A. Maresca

**Affiliations:** a Center for Bioinformatics and Computational Biology, University of Delaware, Newark, Delaware, USA; b Department of Civil and Environmental Engineering, University of Delaware, Newark, Delaware, USA; c Sequencing and Genotyping Center, University of Delaware, Newark, Delaware, USA; d Research Center for Limnology, Indonesian Institute of Sciences, Cibinong, Indonesia; Loyola University Chicago

## Abstract

Here, we report the complete genome sequence of *Microbacterium* sp. strain 10M-3C3, which was isolated from Lake Matano, Indonesia. The genome is 3,387,846 bp long, encodes 3,351 predicted proteins, and has a G+C content of 71.6%.

## ANNOUNCEMENT

*Actinobacteria* frequently dominate microbial assemblages in freshwater lakes, where they are critical to the cycling of carbon and other nutrients (1). After it was observed that nearly 30% of reads in a surface water metagenome from Lake Matano in Indonesia, mapped to *Actinobacteria*, the filtration-acclimation method was used to obtain actinobacterial isolates from this extremely phosphorus-limited lake (2–4). Here, we report the whole-genome sequence of the isolate *Microbacterium* sp. strain 10M-3C3.

**FIG 1:**
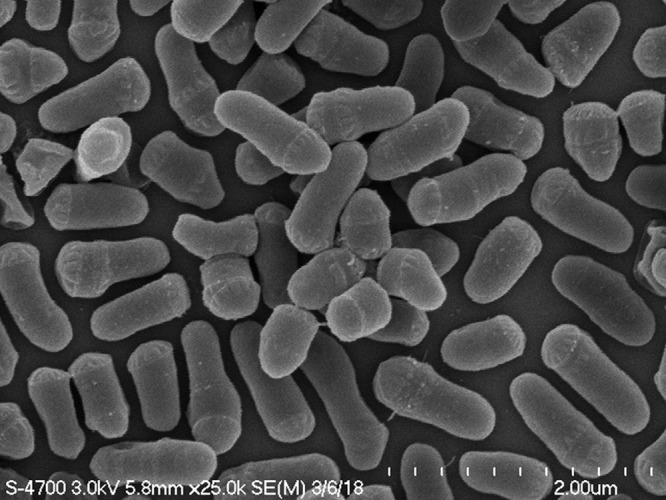
Cells of *Microbacterium* sp. strain 10M-3C3 have a club-shaped (coryneform) morphology. Cells are ∼0.6 to 1.5 µm in length and ∼0.4 µm in diameter when grown in the complex media described here. Cells were fixed in 2% glutaraldehyde, mounted on poly l-lysine-treated silicon support wafers, and then incubated in 1% osmium tetroxide. Samples were then rinsed with Nanopure water, dehydrated in an ethanol dilution series, and dried in a critical point dryer prior to mounting on aluminum stubs and coating with 4.0 nm of platinum with a Leica ACE600 sputter-coater. Imaging was performed on a Hitachi S-4700 FE-SEM at 3.0 kV.

Water was collected from Lake Matano at position 2°28′00″S and 121°17′00″E at a 10-m depth on 5 October 2013 and filtered through a 0.2-µm filter. The filtrate was inoculated into inorganic basal medium and acclimatized to increasing concentrations of nutrient broth-soytone-yeast (NSY) extract medium (3). Following acclimation, liquid cultures were transferred to 0.3% NSY agar plates, and single colonies were restreaked to isolation. Strain 10M-3C3 forms circular, shiny, bright yellow colonies. Individual cells are club shaped (Fig. 1) and stain Gram positive. Genomic DNA was extracted from cells grown in liquid NSY medium using previously described methods for freshwater *Actinobacteria* (5).

Single-molecule real-time (SMRT) libraries were prepared using the standard PacBio protocol for 20-kb libraries (Pacific Biosciences). DNA fragments larger than 10 kb were size-selected using BluePippin (Sage Science). The average fragment size of the library was 30 kb measured by a fragment analyzer (Advanced Analytical Technologies, Inc.). Sequencing was completed on a PacBio RS II single-molecule sequencer in one SMRT cell using P6-C4 chemistry with a 6-h movie.

Reads were assembled using Hierarchical Genome Assembly Process 3 (HGAP3) within the SMRT Analysis version 2.3.0 software from Pacific Biosciences with 23-kb seed read fragment lengths. Reads of inserts were filtered by quality 0.8 and read length 1 kb using the PreAssembler filter version 1 protocol. The single resulting contig was plotted against itself using Gepard version 1.4 (6), and the region of overlap was identified, producing a single circular contig 3,387,846 bp long with 407× coverage and a G+C content of 71.6%. This is at the high end of known microbial G+C contents, which range from ∼15 to 75% (7).

Rapid Annotations using Subsystems Technology (RAST) version 2.0 and Prokka version 1.11 were used to predict the open reading frames (8–10). Default Prokka parameters were used; for RAST, the classic RAST defaults were used (annotation scheme, classic RAST; gene caller, RAST; FIGfam version, Release70; automatically fix errors, yes; backfill gaps, yes). A consensus annotation was generated using BEACON version 3.0 (11). This genome encodes 1 16S-23S-5S rRNA operon, 53 tRNAs, and 3,351 protein coding sequences. Using Magic-BLAST (12) (alignment score of >50), the genome was compared to the metagenomic data set derived from water collected at the same site during the same field campaign (13). Of the more than 880,000 reads in the metagenome, 4,870 mapped to the *Microbacterium* sp. strain 10M-3C3 genome at >86% identity (93% average identity), covering ∼9.3% of the genome. Since reads mapping to this genome represent ∼0.5% of the metagenomic data set, this isolate is representative of the *Microbacterium* spp. in Lake Matano.

### Data availability.

The complete assembled, annotated genome sequence of *Microbacterium* sp. strain MWH-10M3C3 has been deposited in the DDBJ/ENA/GenBank database under the accession no. CP034245. The raw sequence reads have been deposited in the SRA database under accession no. SRX4932182.
